# One generation apart: Individual income and life expectancy in two Swedish cohorts born before and after the expansion of the welfare state

**DOI:** 10.1177/14034948241246433

**Published:** 2024-04-16

**Authors:** Klara Gurzo, Johan Rehnberg, Pekka Martikainen, Olof Östergren

**Affiliations:** 1 Department of Public Health Sciences, Stockholm University, Stockholm, Sweden; 2 Aging Research Center, Karolinska Institutet, Solna, Sweden; 3 Population Research Unit, University of Helsinki, Helsinki, Finland; 4 Center for Social Inequalities in Population Health, Max Planck – University of Helsinki, Helsinki, Finland

**Keywords:** Life expectancy, mortality, cohort analysis, income, social inequalities, welfare state, gender, Sweden

## Abstract

**Aims:**

Social inequalities in mortality persist or even increase in high-income countries. Most evidence is based on a period approach to measuring mortality – that is, data from individuals born decades apart. A cohort approach, however, provides complementary insights using data from individuals who grow up and age under similar social and institutional arrangements. This study compares income inequalities in cohort life expectancy in two Swedish cohorts, one born before and one born after the expansion of the welfare state.

**Methods:**

Data on individuals born in Sweden in 1922–1926 and 1951–1955 were obtained from total population registries. These data were linked to individual disposable income from 1970 and 1999 and mortality between 50 and 61 years of age in 1972–1987 and 2001–2016, respectively. We calculated cohort temporary life expectancies in the two cohorts by income and gender.

**Results:**

Life expectancy, income, and income inequalities in life expectancy increased between the two cohorts, for both men and women. Women born in 1922–1926 had modest income differences in life expectancy, but pronounced differences emerged in the cohort born in 1951–1955. Men with low incomes born in 1951–1955 had roughly similar life expectancy as those with low incomes born in 1922–1926.

**Conclusions:**

Compared with a period approach to life expectancy trends, the cohort approach highlights the stagnation of mortality at the lowest income groups for men and the rapid emergence of a mortality gradient for women. Future research on health inequalities in welfare states should consider underlying factors both from a cohort and period perspective.

## Introduction

Over the past 50 years, the increase in longevity in high-income countries has been socially patterned [1]. People in higher socioeconomic positions [2–6] have experienced a greater increase in longevity than people in lower socioeconomic positions. This difference is also seen in Sweden, where strong redistributive and universalistic welfare policies were gradually established during the second half of the 20th century [4–6].

Studies on social inequalities in mortality typically focus on period life expectancy or age-adjusted mortality rates. Period-based summary measures of mortality are calculated from age-specific mortality risks of individuals from different birth cohorts. As these individuals are often born several decades apart, they are likely to have experienced different social and economic circumstances throughout their lives. The period life expectancy, for example, does not apply to any real birth cohort [7] but instead describes a hypothetical cohort experiencing age-specific mortality rates observed in a single period [8]. Consequently, period measures emphasise factors that influence everyone at the same time such as the COVID-19 pandemic. In 2020, the period life expectancy in Sweden decreased substantially, but the cohort life expectancy only showed modest changes [9].

Cohort measures of mortality, however, are calculated from mortality risks of individuals born at the same time. Hence, cohort measures reflect the mortality rates of individuals who grow up and age under similar social and economic conditions. Unlike the period life expectancy, which can be derived from data collected in a single year, cohort life expectancy requires data from several years or even decades. Cohort measures emphasise factors that may affect individuals differently depending on when they were born. For example, changes in smoking-related mortality are strongly driven by cohort-specific smoking behaviour [1, 10]. Although many studies have found substantial and growing social inequalities related to life expectancy and mortality in high-income countries from a period life expectancy perspective, few studies have examined these conditions from a cohort perspective.

The decades following the Second World War witnessed significant social, economic, and political development in Sweden. Notably, steps were taken to establish a welfare state [11]. Various measures were introduced to provide adequate and affordable housing, support employment, initiate income transfer schemes [11], and create a more comprehensive compulsory educational system [12]. Concurrently, the participation of women in the labour force expanded at an unprecedented pace, primarily among young married women with small children [13, 14]. The share of married working-age women in employment increased from around 10% in the 1940s to more than 80% at the end of the century [14]. In addition, the post-war period was characterised by large economic development and the substantial growth of real wages [15]. The life course health development framework posits that health trajectories depend on the timing and duration of exposure to specific influences, and award a central role to early life development [16]. The post-war welfare state and economic growth may therefore have had a greater impact on individuals who were born after the Second World War, compared with those who were born earlier and were children before these changes occurred.

This study compares income inequalities in cohort temporary life expectancy for people aged 50 to 61 years old between two Swedish cohorts: people born between 1922 and 1926 and people born between 1951 and 1955. People born between 1922 and 1926 experienced a period of instability in Europe and reached adulthood before the establishment of the welfare state. In contrast, people born between 1951 and 1955 grew up when the welfare state was expanding, in a period of relative stability and increasing prosperity. Given the increase in the participation of women in the labour force during this period, we focus on gender-specific patterns and differences.

## Materials and methods

**Figure 1.**
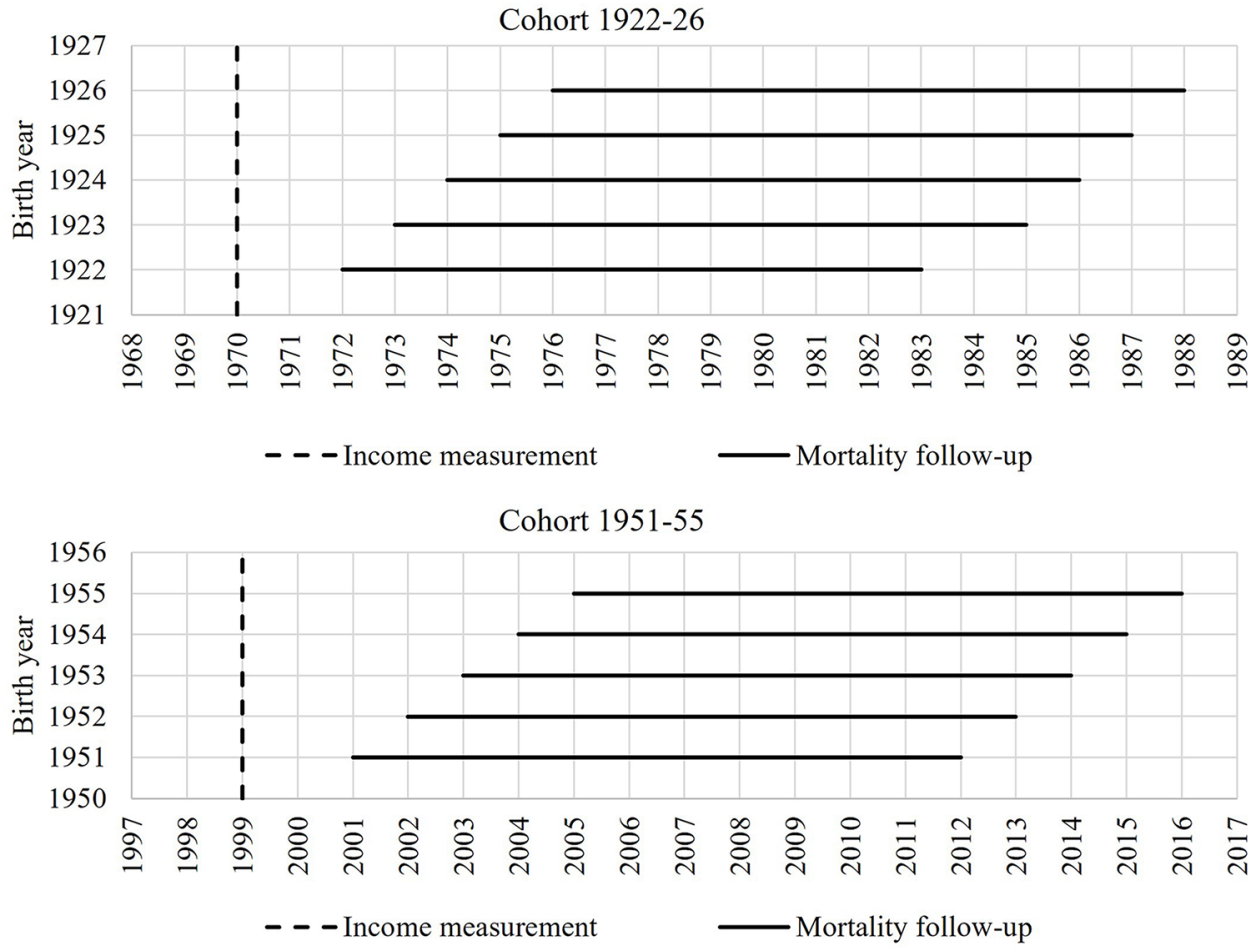
Data structure for income measurement and mortality follow-up by birth year.

The data for this study come from the Swedish Total Population Register and the Income and Tax Register. We specify two birth cohorts: the earlier born cohort consists of individuals born between 1922 and 1926 (cohort 1922–1926) and the later born cohort born between 1951 and 1955 (cohort 1951–1955). We included only individuals born in Sweden who survived until the age of 50 years according to the Total Population Register and who were employed or had a positive income according to the Income and Tax Register. We observed mortality between the ages of 50 and 61 years by specifying the mortality follow-up in cohort 1922–1926 from 1972 to 1983 for those who were born in 1922, and from 1976 to 1987 for those born in 1926. In cohort 1951–1955, mortality follow-up was from 2001 to 2012 for those who were born in 1951, and from 2005 to 2016 for those born in 1955. The same method applies within the two cohorts for all birth years, respectively (Figure 1).

We determined individual disposable income using data from the Income and Tax Register for 1970 and 1999. These comprise all post-tax income, including income from work, transfer payments and capital. We categorised income into vigintiles by sex and cohort. There are some differences in the precise definition of the income variables due to differences in the taxation systems in 1970 and 1999, although these are unlikely to alter the relative income positions of cohort members. In addition, we estimated the absolute income level of each vigintile by adjusting for inflation using the consumer price index with 2020 as the index year, and by calculating the within-vigintile mean income.

We calculated cohort temporary life expectancy (CTLE) for each cohort, sex, and income vigintile group based on observed deaths and person-years of exposure, censoring observations at death, migration or end of follow-up, whichever occurred first. CTLE, expressed as (*τ*) = *E*[min(*T*, *τ*)] (where *T* is the survival time and τ is a specified time point), indicates the average survival time or life expectancy of individuals within [0, *τ*]. Considering censoring, the CTLE can be estimated as the area under the survival curve to a specified time point *τ* [17]. In epidemiological studies, temporary life expectancy is sometimes referred to as partial life expectancy or restricted mean survival time [18]. In this context, the CTLE represents the observed average number of years lived between the ages of 50 and 61 years for the two cohorts. In this study, the theoretical maximum CTLE is 12 years in a scenario in which everyone in the cohort survived to the end of the follow-up.

## Results

The study population comprised 387,866 individuals in cohort 1922–1926 and 489,208 individuals in cohort 1951–1955 (Table I). The mean income was higher for both men and women in cohort 1951–1955. The CTLE between ages 50 and 61 years was 11.5 years for men and 11.7 years for women in cohort 1922–1926 and 11.7 for men and 11.8 years for women in cohort 1951–1955 (Table II). The larger increase for men between the two cohorts led to a less pronounced sex difference in CTLE in cohort 1951–1955.

**Table I.** Descriptive statistics of cohort 1922–1926 and cohort 1951–1955.

	Cohort 1922–1926		Cohort 1951–1955	
	Men (*n*=221,484)	Women (*n*=166,382)	Men (*n*=248,610)	Women (*n*=240,598)
Income^a^ (mean, (SD))	181 (125)	86 (57)	253 (646)	192 (176)
Deceased between ages 50 & 61 years (*n*, (%))	21,640 (9.77)	8,627 (5.19)	13,352 (5.37)	8,842 (3.68)

a Consumer price index adjusted annual individual disposable income in 2020 SEK, from year 1970 for cohort 1922-1926 and 1999 for cohort 1951-1955. 1000s of SEK.

SD: standard deviation.

**Table II.** Cohort temporary life expectancy between ages 50 and 61 years of cohort 1922–1926 and cohort 1951–1955.

	Cohort 1922–1926			Cohort 1951–1955		
	CTLE	95% CI		CTLE	95% CI	
Men	11.51	11.50	11.51	11.73	11.72	11.73
Women	11.73	11.73	11.74	11.81	11.81	11.82

CI: confidence interval; CTLE: cohort temporary life expectancy.

**Figure 2.**
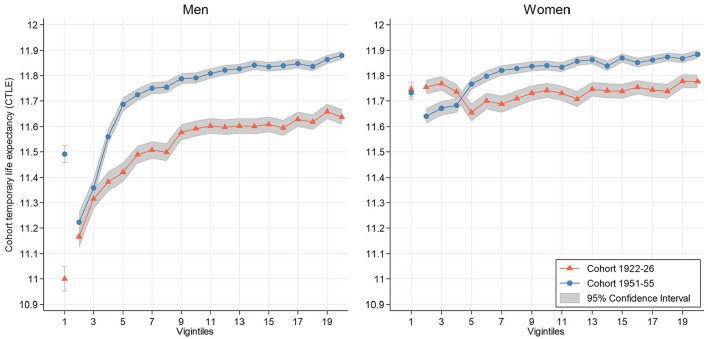
Cohort temporary life expectancy based on individual disposable income vigintiles between ages 50 and 61 years by sex and cohort. The lowest vigintiles are presented as separate data points.

Income differences in life expectancy are presented by plotting CTLE against income vigintiles (Figure 2). We present the lowest vigintile as a separate data point as there are uncertainties surrounding the accuracy of very low incomes, especially in cohort 1951–1955 (see methodological considerations). Both cohorts exhibited a clear income gradient in mortality for men. However, for women, the income–mortality gradient by individual income was present only in cohort 1951–1955. In addition, the change in life expectancy was smaller at the lower end than at the higher end of the income distribution for both men and women, resulting in more pronounced inequalities in cohort 1951–1955. In particular, for men, the rise in CTLE gradually increased up until around the fifth vigintile (corresponding to the 25th percentile) and remained relatively stable. Cohort 1951–1955 had higher CTLE only above the fourth vigintile for women, and the increase in life expectancy between the cohorts was generally smaller for women than for men.

**Figure 3.**
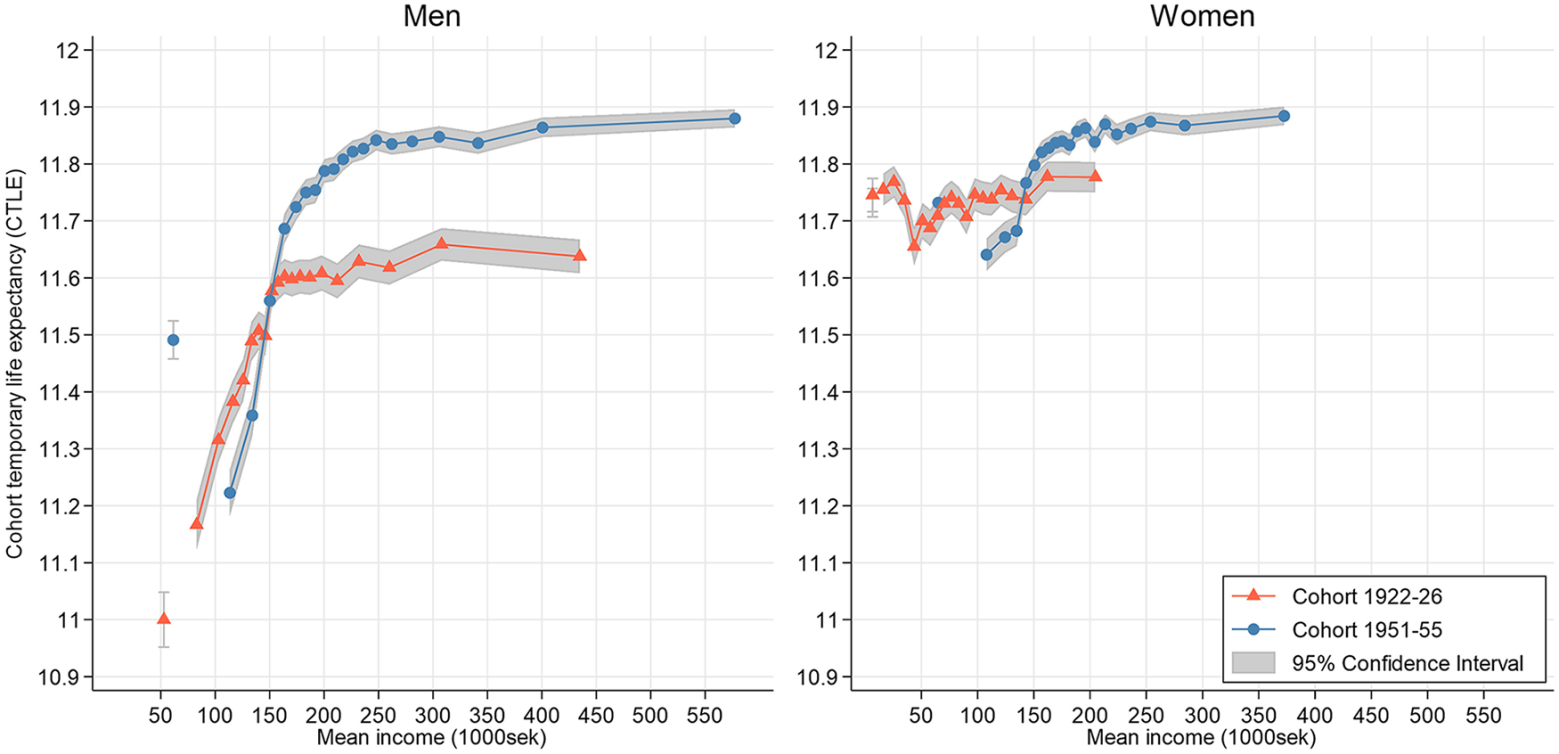
Cohort temporary life expectancy based on within-vigintile mean individual disposable income between ages 50 and 61 years by sex and cohort. The lowest vigintiles are presented as separate data points.

Figure 3 presents the same CTLE on the *y*-axis but changes the scale of the *x*-axis to reflect the mean income in each vigintile. This approach illustrates the level of mortality relative to the absolute income level in both cohorts. Men in the lower part of the income distribution (i.e. an annual income of 100,000–150,000 SEK) had approximately the same life expectancy in both cohorts. This group was a smaller part of cohort 1951–1955 and corresponded to a lower relative income position, as the absolute level of income was higher in cohort 1951–1955 compared with cohort 1922–1926. At higher incomes, the life expectancy of men with the same absolute income increased in cohort 1951–1955. Women in cohort 1951–1955 had a substantial increase in the level of absolute income. Women with an individual income of about 100,000–140,000 SEK had lower life expectancy in cohort 1951–1955 than in cohort 1922–1926, whereas women with higher incomes had a higher life expectancy in cohort 1951–1955.

Household level income might better reflect available economic and material resources compared with individual level income, particularly among women who do not participate in the labour market. Therefore, we also use the sum of both spouses’ incomes after taxes from the 1970 Census and disposable household level income from the 1999 Income and Tax Register. Supplemental Figure 1 presents life expectancy by vigintiles of household income. Overall, the results from the household income analyses were similar to those for individual income. However, we saw a gradient in women’s CTLE in cohort 1922–1926. In cohort 1951–1955, the CTLE in the first vigintile was lower than in the second vigintile for both men and women.

## Discussion

This study explores how income inequalities in temporary life expectancy changed between two Swedish cohorts – one born between 1922 and 1926 and one born between 1951 and 1955 that experienced distinctly different societal contexts. Although cohort temporary life expectancies between ages 50 and 61 years increased between the two cohorts, the increase was socially disparate. Temporary life expectancies were almost the same or even lower in cohort 1951–1955 at the lower end of the income distribution; however, above the fifth vigintile, temporary life expectancies were consistently higher relative to cohort 1922–1926. Hence, mortality related to income inequalities were more pronounced in cohort 1951–1955. Finally, for women, inequalities in mortality by individual income were only observable in cohort 1951–1955.

These results complement insights into common period approaches to life expectancy changes as this study only compares cohorts born before and after the major social and economic shifts that occurred in Sweden in the post-war period. Similar to previous Swedish studies focusing on income disparity trends in mortality from a period perspective [4–6], we found increasing income inequalities in mortality over time. However, the cohort approach emphasised that mortality stagnated at the lowest income groups for men and revealed the rapid emergence of a mortality gradient by individual income for women. Furthermore, the investigation of cohort life expectancy by mean income uncovered that both the income level and the life expectancy stagnated in a small group of men in cohort 1951–1955 compared with the cohort 1922–1926. Recent studies on educational inequalities in mortality have established similar patterns in either less equal societies [19, 20] or later born cohorts [21, 22]. Our findings underscore the lack of mortality improvements in the most disadvantaged groups even at the time of rapid social and economic development in Sweden during the post-war period.

On the one hand, individuals with low income might have benefitted less from the improvements in educational opportunities, social protection, and material living conditions. On the other hand, income inequalities have increased between the two cohorts [23] potentially exacerbating the disadvantage of behavioural and social risks in the lower socioeconomic groups. The widening absolute and relative income inequalities suggest that absolute levels of income may have a very different meaning with respect to the differences in the standard of living between income groups in the later cohorts. Similarly, disparities in exposure to health-related living conditions (e.g. air pollution, food accessibility, overcrowding, etc.) may have become more concentrated in the lower income groups, constraining behavioural choices. Understanding why the health of disadvantaged individuals did not fare better in the welfare state may be important for understanding health inequalities in the context of the Scandinavian welfare state.

Another contributing explanation for why individuals with low incomes did not experience life expectancy increases could be the increasing role of health selection [24, 25]. During the time between the two cohorts, the educational system became more comprehensive, potentially leading to higher social fluidity [26], which has been suggested to increase selection into socioeconomic position, either by health or determinants of health [27]. Consequently, individuals who can leverage the opportunities provided by the welfare state in terms of higher incomes may over time have become increasingly selected on good health, while individuals with lower incomes may in turn have been negatively selected. Therefore, it is possible that the overall improvements in educational qualifications between the two cohorts may have led to increased marginalisation for those occupying the lowest income positions in cohort 1951–1955.

Finally, important cultural and behavioural changes also took place between the two cohorts, factors that may have contributed to persisting health inequalities in welfare states [25, 27, 28]. Concurrent with the establishment of the welfare state, smoking and alcohol consumption have become increasingly socially patterned [29]. As women entered the labour market, gendered patterns of health behaviours also changed. An increase in smoking rates among women tend to emerge alongside female participation in the labour market, and cigarettes have been seen as a symbol of female emancipation [30]. In Sweden, smoking attributable death rates peaked around 1976 for men and in 2015 for women [31]. This finding can contribute both to the fact that we see smaller gender differences in life expectancy in cohort 1951–1955, and also to the emergence of inequalities in mortality by women’s own income in cohort 1951–1955. In our analyses, we cannot directly disentangle the contribution of the aforementioned potential mechanisms leading to increasing mortality inequalities.

### Methodological considerations

Given the comparatively low level of labour force participation among women in cohort 1922–1926, it may be unlikely that their individual incomes reflected their social status. Therefore, previous studies on income and mortality typically consider household income for women, especially for older generations such as in cohort 1922–1926 [5, 6, 29]. In supplemental analyses, we also rank both women and men by household income (Supplemental Figure 1). This approach revealed a gradient in mortality by household income for women in both cohorts. As with men, the gradient was more pronounced in cohort 1951–1955. The fact that the association between individual income and mortality risk emerged in cohort 1951–1955 is indicative of a societal shift in which women’s socioeconomic achievement and labour market participation became more important for their living conditions and, subsequently, health. As women entered the labour market, new processes may have increasingly contributed to the inequalities in mortality, for example, occupational exposures and health-related selection out of the labour market. These conditions may have contributed to the finding that the shape of the income gradient in mortality more closely resembled one another for men and women in cohort 1951–1955 than in cohort 1922–1926, both in terms of household and individual income. Gender differences in how specific income measures are associated with mortality reveal that the disparities among women are more sensitive to measurement specification [32]. Therefore, studies on income inequalities and mortality should consider the historical time, context and specific income measurement.

In cohort 1951–1955, the life expectancy according to individual income in the bottom vigintile was higher than expected for both men and women. Similar findings have been reported elsewhere [6]. It is possible that this can be partly attributed to inaccuracies in administrative data. Unregistered migration, non-taxable incomes, as well as tax evasion can underestimate the level of income for those with a very low level of registered income [6]. In Supplemental Table I, we present the relative size and life expectancies for those with registered income, income in the lowest vigintile, zero income and missing income. These groups differed in size and life expectancy by both gender and cohort, indicating that they are likely not to be comparable between our cohorts. In addition, we can only measure income in one year for the 1922–1926 cohort. In the later cohort we had access to more years. In Supplemental Figure 2, we present the life expectancies by vigintiles based on 5 years of income information (between 1997 and 2001), and find that the results are highly similar to those obtained when measuring income in a single year.

Estimating cohort life expectancies rather than period life expectancies comes with a cost: the application of temporary life expectancies in a relatively narrow age range of between 50 and 61 years. The earliest time we had income data was 1970 and the latest time we could observe mortality was 2016. Previous evidence indicates that social inequalities in mortality are large around this age range [33], suggesting that our results are less representative of old age mortality when avoidable deaths are less common. Future studies, presumably with access to more data, could use a longer follow-up and incorporate causes of death.

## Conclusions

This study provides the first evidence that social inequalities in adult mortality were not smaller in cohorts that grew up in Sweden after the expansion of the welfare state compared with cohorts who only experienced the welfare state as adults. Women’s own income was only related to life expectancy in cohort 1951–1955, when women experienced higher levels of labour force participation. Cohort life expectancy was consistently higher in cohort 1951–1955 for men and women with higher incomes, but the life expectancy of men and women with very low incomes born in 1951–1955 was strikingly similar to men and women with low incomes born in 1922–1926. The cohort approach highlights the importance of the lack of mortality improvements in the most disadvantaged groups when trying to understand the persistence of health inequalities in egalitarian welfare states.

## Supplemental Material

sj-docx-1-sjp-10.1177_14034948241246433 – Supplemental material for One generation apart: Individual income and life expectancy in two Swedish cohorts born before and after the expansion of the welfare state Supplemental material, sj-docx-1-sjp-10.1177_14034948241246433 for One generation apart: Individual income and life expectancy in two Swedish cohorts born before and after the expansion of the welfare state by Klara Gurzo, Johan Rehnberg, Pekka Martikainen and Olof Östergren in Scandinavian Journal of Public Health
