# Supplementary material for: One generation apart: Individual income and life expectancy in two Swedish cohorts born before and after the expansion of the welfare state
Source: Scand J Public Health. 2024 Apr 16;53(4):351–8. doi: 10.1177/14034948241246433 (PMC12048735; doi:10.1177/14034948241246433)
Supplement: sj-docx-1-sjp-10.1177_14034948241246433 – Supplemental material for One generation apart: Individual income and life expectancy in two Swedish cohorts born before and after the expansion of the welfare state [file sj-docx-1-sjp-10.1177_14034948241246433.docx]

Supplementary file

Supplementary figure 1 Cohort temporary life expectancy based on household level income vigintiles between age 50 and 61 by sex and cohort. The lowest vigintiles are presented as separate data points

| Supplementary Table 1. Cohort temporary life expectancy and composition of individuals reporting zero, missing, any income, or income in the lowest vigintile by cohort and gender | | | | | | | | |
| --- | --- | --- | --- | --- | --- | --- | --- | --- |
|  | **Cohort 1922-26** | | | | | | | |
|  | **Men** (N=232,556) | | | | **Women** (N=229,526) | | | |
|  | CTLE^1^ | | Size | | CTLE^1^ | | Size | |
|  | Mean | Diff^2^ | n | %^3^ | Mean | Diff^2^ | n | %^3^ |
| Registered income | 11.51 |  | 221,484 | 0.95 | 11.73 |  | 166,382 | 0.73 |
| First income vigintile | 11.00 | -0.51 | 11,075 | 4.76 | 11.75 | -0.02 | 8,341 | 3.63 |
| Zero income | 10.95 | -0.56 | 7,471 | 3.21 | 11.61 | -0.12 | 10,413 | 4.54 |
| Missing income | 10.87 | -0.64 | 3,601 | 1.55 | 11.70 | -0.03 | 52,731 | 22.97 |
|  |  |  |  |  |  |  |  |  |
|  | **Cohort 1951-55** | | | | | | | |
|  | **Men** (N=256,078) | | | | **Women** (N=247,858) | | | |
|  | CTLE^1^ | | Size | | CTLE^1^ | | Size | |
|  | Mean | Diff^2^ | n | %^3^ | Mean | Diff^2^ | n | %^3^ |
| Registered income | 11.73 |  | 248,610 | 0.97 | 11.81 |  | 240,598 | 0.97 |
| First income vigintile | 11.54 | -0.19 | 12,437 | 4.86 | 11.76 | -0.05 | 12,063 | 4.87 |
| Zero income^4^ | 11.63 | -0.10 | 2,386 | 0.93 | 11.74 | -0.07 | 1,122 | 0.45 |
| Missing income | 11.94 | 0.19 | 5,082 | 1.99 | 11.98 | 0.17 | 6,138 | 2.47 |
| ^1^ Cohort temporary life expectancy between ages 50 and 61  ^2^ Difference from registered income  ^3^ Gender-specific proportions  ^4^ Zero and negative income values in the register were set to zero. | | | | | | | | |

Supplementary figure 2. Cohort temporary life expectancy based on individual level income vigintiles between age 50 and 61 by sex and cohort (for cohort 1951-55 both one year and five year-based income vigintiles are presented). The lowest vigintiles are presented as separate data points.
